# Supplementary material for: The Gut Bacteria Dysbiosis Contributes to Chronic Graft-Versus-Host Disease Associated With a Treg/Th1 Ratio Imbalance
Source: Front Microbiol. 2022 Sep 8;13:813576. doi: 10.3389/fmicb.2022.813576 (PMC9493085; doi:10.3389/fmicb.2022.813576)
Supplement: Supplementary file 1 [file Data_Sheet_1.zip › P101SC18090073-01-B1-3-4_result/05.WebShow/taxa_charts_html/charts/zW72PzAuNhHoCkCcbo2EDFzehZcaep_legend.pdf]

- k__Bacteria;Other;Other;Other
- k__Bacteria;p__Actinobacteria;c__unidentified_Actinobacteria;o__Bifidobacteriales
- k__Bacteria;p__Actinobacteria;c__unidentified_Actinobacteria;o__Micrococcales
- k__Bacteria;p__Bacteroidetes;c__Bacteroidia;o__Bacteroidales
- k__Bacteria;p__Bacteroidetes;c__Bacteroidia;o__Flavobacteriales
- k__Bacteria;p__Firmicutes;Other;Other
- k__Bacteria;p__Firmicutes;c__Bacilli;o__Lactobacillales
- k__Bacteria;p__Firmicutes;c__Clostridia;o__Clostridiales
- k__Bacteria;p__Firmicutes;c__Erysipelotrichia;o__Erysipelotrichales
- k__Bacteria;p__Firmicutes;c__Negativicutes;o__Selenomonadales
- k__Bacteria;p__Fusobacteria;c__Fusobacteriia;o__Fusobacteriales
- k__Bacteria;p__Melainabacteria;Other;Other
- k__Bacteria;p__Proteobacteria;c__Deltaproteobacteria;o__Desulfovibrionales
- k__Bacteria;p__Proteobacteria;c__Gammaproteobacteria;o__Enterobacteriales
- k__Bacteria;p__Proteobacteria;c__Gammaproteobacteria;o__Pseudomonadales
- k__Bacteria;p__Proteobacteria;c__Gammaproteobacteria;o__unidentified_Gammaproteobacteria
- k__Bacteria;p__Tenericutes;c__Mollicutes;o__
- k__Bacteria;p__Verrucomicrobia;c__Verrucomicrobiae;o__Verrucomicrobiales
- k__Bacteria;p__unidentified_Bacteria;c__unidentified_Bacteria;o__unidentified_Bacteria
- All Other Categories
